# Construction of a risk prediction model for falls in elderly lung cancer patients with sarcopenia

**DOI:** 10.3389/fonc.2025.1533368

**Published:** 2025-06-20

**Authors:** Qing Wang, Xiao Han, Jun Zhang, Mengying Hu, Jiaojiao Xu, Qiongqiong Ai, Hequn Wei, Jiao Yu, Haiping Ma

**Affiliations:** ^1^ The Second Affiliated Hospital of Nanchang University, Nanchang, Jiangxi, China; ^2^ School of Nursing, Jiangxi Medical College, Nanchang University, Nanchang, Jiangxi, China; ^3^ The Affiliated Stomatological Hospital of Nanchang University, Nanchang, Jiangxi, China

**Keywords:** elderly, lung cancer, sarcopenia, falls, predictive model

## Abstract

**Background:**

To explore the risk factors associated with falls in elderly lung cancer patients with sarcopenia, construct a predictive model, and validate its performance.

**Methods:**

This cross-sectional study involved 316 lung cancer patients with sarcopenia who were hospitalized in the oncology, thoracic surgery, and respiratory medicine departments of a tertiary hospital in Jiangxi Province between January 2023 and December 2023. Data were collected through questionnaires and physical measurements. A logistic regression predictive model was developed on the basis of independent risk factors.

**Results:**

The incidence of falls among elderly lung cancer patients with sarcopenia was 19.94%. Multivariate logistic regression analysis identified multiple metastases, nocturia (≥3 times per night), sleep disorders, frailty, and malnutrition as independent risk factors for falls. The Hosmer - Lemeshow test indicated good model fit (X^2^ = 5.353, *P*=0.719), with an overall predictive accuracy of 83.7%. The area under the ROC curve (AUC) was 0.832, and the Youden index reached a maximum of 0.577, corresponding to a sensitivity of 74.7%, specificity of 83.0%, and an optimal cut-off value of 0.221.

**Conclusion:**

The risk prediction model for falls in elderly lung cancer patients with sarcopenia, which is based on independent predictors, demonstrated good predictive performance. This model facilitates the timely identification of high-risk patients, providing scientific evidence to support the development of precise clinical management strategies.

## 1 Introduce

According to the 2020 report by the International Agency for Research on Cancer (IARC), lung cancer ranks second in global cancer incidence. It is the leading cause of cancer-related mortality worldwide, accounting for 11.4% of all diagnosed cancers and 18% of cancer deaths (1). Notably, age is a critical factor in the onset of lung cancer, with approximately 60% of patients being over 65 years old at the time of diagnosis (2). This trend is particularly alarming against the backdrop of the aging of the global population,55 to 79 years old is the high incidence rate of lung cancer age group, accounting for more than 70% of all cases (3).Sarcopenia, also known as muscle wasting syndrome, is a geriatric syndrome closely associated with aging. Its primary characteristics include the progressive loss of skeletal muscle mass, reduced muscle strength, and impaired muscle function (4). Of particular concern is the high prevalence of sarcopenia among lung cancer patients, who are often prone to nutritional and metabolic disorders (5). Studies indicate that the incidence of skeletal muscle loss in lung cancer patients ranges from 46.8% to 55.8% (6, 7). Falls are defined as sudden, involuntary, and unintentional postural changes that result in an individual landing on the ground or a lower plane (8). According to data from the World Health Organization (WHO) (9), approximately 684,000 individuals die each year due to falls, making falls the second leading cause of death from unintentional injuries worldwide, adults aged 60 years and older experience the highest number of fatal falls. Sarcopenia, which affects skeletal muscle mass and strength, weakens the physical condition and function of the elderly. This decline in function leads to a significant reduction in mobility and balance, which is a key factor contributing to falls. Moreover, among fall patients, cancer was the most prevalent underlying disease, with lung cancer accounting for 30.8% of cases (10, 11).Fall risk assessment tools are crucial for identifying and predicting patients at risk of falling. These tools currently encompass four main categories: the fall risk comprehensive assessment scale, the fall-related psychological assessment scale, the balance function scale, and the fall prevention questionnaire (12). The Morse Fall Risk Assessment Scale is commonly used in clinical settings to evaluate patients’ risk of falling and determine their fall risk levels (13). While these scales are widely used in general or elderly patients, they may not accurately assess the fall risk in elderly patients with specific disease backgrounds. In light of these findings, the present study aimed to identify potential risk factors for falls in elderly lung cancer patients with sarcopenia and develop a risk prediction model. The model is visualized via a nomogram to facilitate its application in clinical practice, providing an efficient and user-friendly assessment tool. This tool is intended to assist healthcare professionals in rapidly and accurately identifying high-risk patients, implementing targeted management strategies, reducing the occurrence of falls, and ultimately improving the quality of life of lung cancer patients.

## 2 Participants and methods

### 2.1 Study participants

This study adopted a cross-sectional design and strictly met the inclusion and exclusion criteria. Participants were selected from inpatients admitted to the oncology, thoracic surgery, and respiratory medicine departments of a tertiary hospital in Jiangxi Province between January 2023 and December 2023. The inclusion criteria were as follows: (1) aged ≥60 years (14); (2) diagnosed with primary lung cancer on the basis of histopathology or cytology; (3) positive results (score >10) on the Chinese version of the five-item Sarcopenia SARC-CALF scale (15); and (4) willing to complete physical measurements and questionnaire assessments. The exclusion criteria were as follows: ① severe cognitive impairment preventing effective communication; ② bedridden for extended periods; and ③ edema.

Through a systematic review of relevant literature and discussions within the research group, supplemented by two rounds of expert consultations conducted via letters, the researchers developed a questionnaire to assess fall risk factors in elderly lung cancer patients with sarcopenia. The questionnaire comprises 38 items. In accordance with the Kendall Sample Size criteria, the sample size should be 5 to 10 times the number of variables to ensure the accuracy and reliability of the study (16).A total of 38 study variables were identified on the basis of prior literature reviews and expert consultations. The required minimum sample size was calculated to be at least 190 participants. To account for potential nonresponse during data collection and to ensure sample representativeness and study accuracy, the sample size was increased by 20%, resulting in a minimum of 228 participants. According to the EPV (Events Per Variable) principle for logistic regression (17), at least 10 events per variable are required for robust results. Given that 4–6 predictors were estimated for the final model, a minimum of 40–60 participants with fall events was necessary for the analysis. Ultimately, 316 elderly lung cancer patients with sarcopenia were included in the study, 63 of whom experienced falls. The sample size met all the statistical requirements.

### 2.2 Survey tools

#### 2.2.1 Fall risk factor questionnaire for elderly lung cancer patients with sarcopenia

The survey questionnaire primarily covers six key domains: sociodemographic characteristics, disease - related factors, physical health, medication use, functional and activity status, and laboratory indicators. The following section provides explanations for some of the assessment methods used:

1. The visual analogue scale (VAS) (18) was used to evaluate cancer pain. This scale quantifies patients’ pain perception via a 10 cm horizontal line. The left endpoint is marked as 0, representing “no pain,” and the right endpoint is marked as 10, representing “severe pain.” Patients pinpoint their perceived pain level on the line. Scores range from 0 to 10, with 0 indicating no pain, 1–3 indicating mild pain, 4–6 indicating moderate pain, and 7–10 indicating severe pain.
2. The Barthel Index (BI) (19), developed by Mahoney and Barthel in 1965 and translated into Chinese by Hou Dongzhe in 2012 (Cronbach’s α=0.916), was used to assess activities of daily living (ADL). It includes 10 core items covering eating, grooming, dressing, toileting, and mobility. The total scores range from 0 to 100, with lower scores indicating greater dependency. Dependency levels are categorized as severe (≤40), moderate (41–60), mild (61–99), and complete independence (100).
3. The Mini Nutritional Assessment Short Form (MNA-SF) (20), originally developed by Guigoz et al., evaluates the nutritional status of elderly individuals across six dimensions: food intake, weight change, mobility, psychological stress and acute illness, and body mass index (BMI). The scores for each dimension range from 0–3, with a total score ranging from 0–14. Nutritional status is categorized as normal (12–14), at risk of malnutrition (8–11), or malnourished (0–7).
4. Frailty status was assessed via the FRAIL scale (21), which was proposed in 2008 by an international group of experts on nutrition, health, and aging. This scale is designed to screen frailty in the clinical population of elderly individuals. It consists of five items: self-reported fatigue, reduced physical activity, unintentional weight loss of ≥5% within one year, diminished endurance or increased resistance, and the presence of five or more chronic diseases. Scores of 0–2 indicate no frailty or prefrailty, whereas scores of 3–5 indicate frailty. (Cronbach’s α=0.705).
5. Gait speed was assessed via the 6-meter walk test recommended by the Asian Working Group for Sarcopenia (AWGS) (22). A 12-meter straight line was marked on a flat surface, with clear indicators at the starting point, 3 m, 9 m, and the endpoint. The participants walked along the line; timing began at 3 m and ended at 9 m. The test was repeated three times, with the fastest time recorded. A gait speed <1.0 m/s was considered reduced, whereas a speed ≥1.0 m/s was considered normal.
6. Grip strength was measured via an EH101 digital dynamometer from Xiangshan Weighing Equipment Group, with measurements in kilograms (kg). For safety and accuracy, particularly for patients with peripherally inserted central catheters (PICCs), only the dominant hand was tested without exerting pressure on the catheter. The participants squeezed the dynamometer with maximum strength for 3 seconds, with two trials separated by a 30-second rest. The highest value was recorded. According to the AWGS standards, a grip strength <28 kg in men and <18 kg in women indicates reduced strength (22).
7. Stride length, defined as the vertical distance between consecutive heel contacts of the same foot, was measured in centimeters. The participants were instructed to walk naturally, and measurements were recorded.

#### 2.2.2 Modified sarcopenia screening questionnaire

The SARC-CalF is a sarcopenia screening tool recommended by the Asian Working Group for Sarcopenia. Developed by Barbosa-Silva in 2016 (23) as an extension of the SARC-F scale, it incorporates calf circumference (CC) as a critical objective measure. The CC cut-off is set at 34 cm for men and 33 cm for women. In the SARC-CalF scale, CCs ≤34 cm for men or ≤33 cm for women score 10 points, whereas larger circumferences score 0 points. A total score ≥11 indicates sarcopenia screening positivity. A Chinese version of the SARC-CalF was applied to cancer patients by Fu et al. (24) The area under the receiver operating characteristic (ROC) curve was 0.75, indicating high screening efficiency. In this study, trained researchers used nonelastic measuring tapes to measure the circumference at the widest point of the nondominant calf.

### 2.3 Data collection methods

After approval was obtained from the data collection institution, elderly lung cancer patients with sarcopenia hospitalized in the oncology, thoracic surgery, and respiratory medicine departments of a tertiary hospital in Jiangxi Province were conveniently sampled according to the inclusion and exclusion criteria. Two nursing graduate students were selected as investigators and received standardized training to ensure measurement consistency. A total of 327 questionnaires were distributed and returned, yielding a 100% response rate. After excluding 11 invalid questionnaires due to incomplete answers or evident uniformity in responses, 316 valid questionnaires were obtained, with an effective response rate of 96.64%.

### 2.4 Statistical methods

The collected data were sequentially numbered and preliminarily organized via Excel 2003. After dual-person verification, the data were entered into an SPSS database. Statistical description and univariate and multivariate analyses were conducted via SPSS 27.0. A fall risk prediction model was constructed and validated via R language version 4.3.2. Statistical tests were performed via two-sided tests, with a significance level of α=0.05. A *P* value <0.05 was considered to indicate statistical significance.

## 3 Results

### 3.1 General characteristics of the participants and fall incidence

The study ultimately included 316 elderly lung cancer patients with sarcopenia who met the inclusion and exclusion criteria. Among them, 63 patients experienced falls, resulting in a fall rate of 19.94%. The training set included 221 patients, with 47 experiencing falls (fall rate: 21.27%), whereas the validation set included 95 patients, with 16 experiencing falls (fall rate: 16.84%) See **Table 1**.

**Table 1:** Univariate analysis of fall risk factors in the training set (n = 221).

Variable	Total (n = 221)	Nonfall Group (n = 174)	Fall Group (n = 47)	*X*2*/F/T*	*P*
Mode of Admission				15.211^2)^	<0.001
- Walking	190 (85.97)	158 (90.80)	32 (68.09)		
- Assisted	16 (7.24)	9 (5. 17)	7 (14.89)		
- Wheelchair	12 (5.43)	6 (3.45)	6 (12.77)		
- Stretcher	3 (1.36)	1 (0.57)	2 (4.26)		
Bone Metastasis				5.901^1)^	0.015
- No	150 (67.87)	125 (71.84)	25 (53. 19)		
- Yes	71 (32. 13)	49 (28. 16)	22 (46.81)		
Multiple Metastases				5.472^1)^	0.019
- No	136 (61.54)	114 (65.52)	22 (46.81)		
- Yes	85 (38.46)	60 (34.48)	25 (53.19)		
Diabetes				4.209^1)^	0.04
- No	186 (84. 16)	151 (86.78)	35 (74.47)		
- Yes	35 (15.84)	23 (13.22)	12 (25.53)		
Frailty Score				17.737^1)^	<0.001
- No frailty	54 (24.43)	48 (27.59)	6 (12.77)		
- At risk of frailty	114 (51.58)	95 (54.60)	19 (40.43)		
- Frailty syndrome	53 (23.98)	31 (17.82)	22 (46.81)		
Nutritional Score				18.077^1)^	<0.001
- Normal nutrition	59 (26.7)	53 (30.46)	6 (12.77)		
- At risk of malnutrition	128 (57.92)	103 (59.20)	25 (53. 19)		
- Malnourished	34 (15.38)	18 (10.34)	16 (34.04)		
Nocturia (≥3 times)				7.181^1)^	0.007
- No	165 (74.66)	137 (78.74)	28 (59.57)		
- Yes	56 (25.34)	37 (21.26)	19 (40.43)		
Sleep Disorders				18.229^1)^	<0.001
- No	143 (64.71)	125 (71.84)	18 (38.30)		
- Yes	78 (35.29)	49 (28. 16)	29 (61.70)		
Constipation				8.219^1)^	0.004
- No	151 (68.33)	127 (72.99)	24 (51.06)		
- Yes	70 (31.67)	47 (27.01)	23 (48.94)		
BI Index				21.519^1)^	<0.001
- Independent	62 (28.05)	56 (32. 18)	6 (12.77)		
- Mild dependence	117 (52.94)	95 (54.60)	22 (46.81)		
- Moderate dependence	32 (14.48)	19 (10.92)	13 (27.66)		
- Severe dependence	10 (4.52)	4 (2.30)	6 (12.77)		
Gait				12.671^1)^	0.002
- Normal gait	175 (79. 19)	146 (83.91)	29 (61.70)		
- Unstable gait	39 (17.65)	25 (14.37)	14 (29.79)		
- Imbalanced gait	7 (3. 17)	3 (1.74)	4 (8.51)		
Gait Speed	0.80± 0.217	0.830± 0.201	0.69 ± 0.24	3.922^3)^	<0.001
Grip Strength	24.36± 6.30	24.81 ± 6.15	22.71 ± 6.60	2.043^3)^	0.042
Albumin	35.858 ± 5.254	36.304± 5.258	34.2094.950	2.452^3)^	0.015

^1)^X^2^ value.

^2)^Fisher’s exact test.

^3)^T value.

### 3.2 Univariate analysis results

Univariate analysis of the training set revealed statistically significant differences between the fall group and nonfall group in 14 variables: admission type, bone metastasis, multiple metastases, diabetes, nocturia frequency ≥3 times, sleep disorders, constipation, activities of daily living (ADL) score, frailty score, nutritional score, grip strength, gait, gait speed, and albumin levels.

### 3.3 Multicollinearity analysis of fall-related factors in the training set

Examination of the variables revealed no significant multicollinearity issues, allowing further multivariate logistic regression analysis. The coding methods are shown in **Table 2**. Multivariate logistic regression analysis revealed that multiple metastases (OR=2.628, 95% CI: 1.174–5.883), nocturia frequency ≥3 times (OR=2.651, 95% CI: 1.180–5.955), sleep disorders (OR=3.328, 95% CI: 1.474–7.513), frailty (OR=6.372, 95% CI: 1.788–22.708), and malnutrition (OR=5.244, 95% CI: 1.531–17.961) were independent risk factors for falls in elderly lung cancer patients with sarcopenia (*P*<0.05). See **Table 3**.

**Table 2:** Variable assignment.

Variable Name	Assignment Description
Mode of Admission	Walking = 0; Assisted = 1; Wheelchair = 2; Stretcher = 3 (dummy variable)
Bone Metastasis	No = 0; Yes = 1
Multiple Metastases	No = 0; Yes = 1
Diabetes	No = 0; Yes = 1
Nocturia (≥3 times)	No = 0; Yes = 1
Sleep Disorders	No = 0; Yes = 1
Constipation	No = 0; Yes = 1
BI Index	Independent = 0; Mild dependence = 1; Moderate dependence = 2;Severe dependence = 3 (dummy variable)
Frailty Score	No frailty = 0; Prefrailty = 1; Frailty = 2 (dummy variable)
MNA-SF Score	Normal nutrition = 0; At risk of malnutrition = 1; Malnourished = 2 (dummy variable)
Grip Strength	Input as the raw value
Gait	Normal gait = 0; Unstable gait = 1; Imbalanced gait = 2 (dummy variable)
Gait Speed	Input as the raw value
Albumin	Input as the raw value

The following 14 variables that showed significant differences in univariate analysis were assigned values: Mode of admission, bone metastasis, multiple metastases, diabetes, frailty score, nutritional Score, nocturia(≥3 times), sleep disorders, constipation, BI index, gait, gait speed, grip strength, and albumin.

**Table 3:** Multivariate analysis results in the training set.

Variable	*β*	*SE*	*Wald*	*P*	*OR*	*95%CI*
Constant	-4.1	0.759	29.16	0	0.017	
Multiple Metastases	0.966	0.411	5.518	0.019	2.628	1.174-5.883
Nocturia (≥3 times)	0.975	0.413	5.577	0.018	2.651	1.180-5.955
Sleep Disorders	1.202	0.415	8.378	0.004	3.328	1.474-7.513
Frail Score						
- No frailty (0 points)			11.862	0.003		(Reference)
- Prefrailty (1–2 points)	0.591	0.62	0.909	0.34	1.806	0.536-6.087
- Frailty (≥3 points)	1.852	0.648	8.16	0.004	6.372	1.788-22.708
MNA-SF Score						
- Normal nutrition (12–14)			8.769	0.012		(Reference)
- Risk of malnutrition (8–11)	0.373	0.537	0.482	0.487	1.452	0.507-4.161
- Malnutrition (0–7)	1.657	0.628	6.961	0.008	5.244	1.531-17.961

*β*, Beta Coefficient; *SE*, Standard Error; *Wald*, Wald Statistic; *P*, *P*-value, *P<0.05* suggests the variable significantly predicts fall risk. OR, Odds ratio. 95%CI, 95% Confidence Interval. Frailty was assessed using the FRAIL scale, and nutritional status was evaluated via the Mini Nutritional Assessment Short Form (MNA-SF). Both frailty and nutritional status are unordered multicategorical variables and cannot be directly included in multivariate analysis without appropriate coding. Therefore, dummy variable coding was applied, with the following reference groups: Frailty Score: Reference group ="No frailty (0 points).", MNA-SF Score: Reference group = "Normal nutrition (12–14 points).")

### 3.4 Construction of the fall risk prediction model for elderly lung cancer patients with sarcopenia

**Figure 1:**
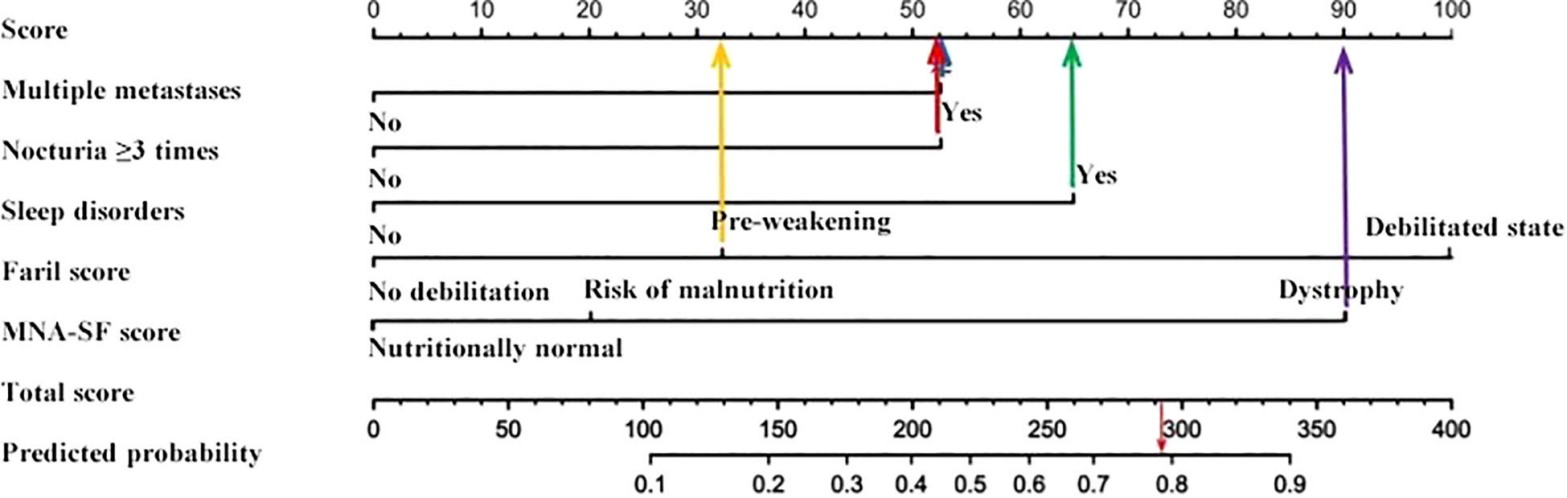
Column line diagram.

On the basis of the multivariate logistic regression results, five risk factors—multiple metastases, nocturia frequency ≥3 times, sleep disorders, frailty score, and malnutrition score—were included in the fall risk prediction model. The model was visualized via a nomogram generated in R software, as shown in **Figure 1**.

### 3.5 Validation and evaluation of the fall risk prediction model

**Figure 2:**
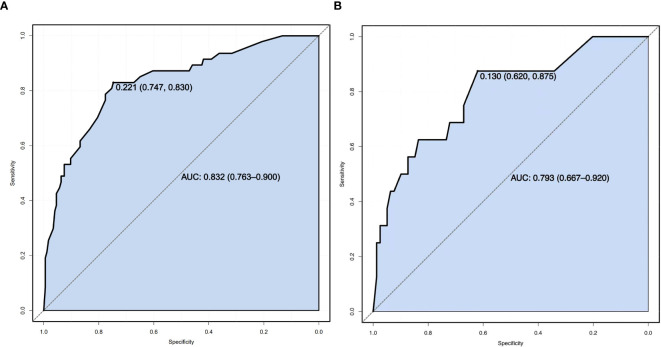
**(A)** Training set ROC Curve. **(B)** ROC Curve of the validation set.ROC Curve (Receiver Operating Characteristic Curve): A graphical tool used to evaluate the performance of a binary classification model. It plots the relationship between the True Positive Rate (TPR) and the False Positive Rate (FPR) across varying classification thresholds, reflecting the model’s ability to distinguish between positive and negative samples. The closer the curve is to the top-left corner, the better the model’s performance. The x-axis represents the False Positive Rate (FPR, calculated as 1−Specificity), and the y-axis represents the True Positive Rate (TPR, equivalent to Sensitivity).

**Figure 3:**
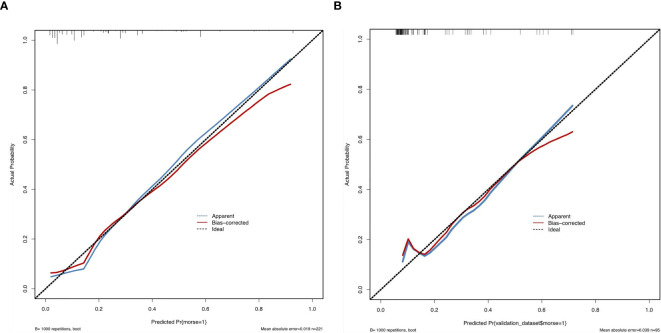
**(A)** Calibration of the training set. **(B)** Calibration of the validation set. Calibration refers to the consistency between the predicted probabilities of a model and the actual observed frequencies of outcomes. It reflects the reliability of the model’s predicted probabilities. As illustrated in the figure, the calibration curve closely follows the ideal diagonal reference line, indicating strong agreement between the predicted probabilities and the observed frequencies. This further validates the model’s predictive accuracy and reliability. The x-axis represents the model’s predicted probabilities (ranging from 0% to 100%), while the y-axis represents the actual observed event frequencies.

**Figure 4:**
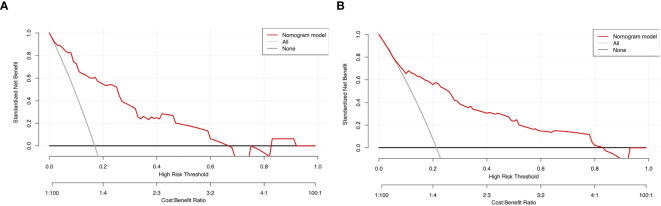
**(A)** Training set decision curve. **(B)** Validation set Decision Curve. Decision Curve Analysis (DCA): A graphical evaluation tool used to quantify the clinical utility of predictive models. It visually demonstrates the clinical net benefit across varying threshold probabilities, aiding decision-makers in selecting optimal intervention strategies. In the figure: The black horizontal line represents an extreme scenario (“Treat None” strategy), where the clinical net benefit is zero. The gray diagonal line represents another extreme scenario (“Treat All” strategy), where the clinical net benefit decreases gradually as the threshold probability increases, exhibiting a negative slope. The red curve depicts the actual DCA decision curve. The red curve lies above both extreme lines across most threshold probability ranges, indicating that our predictive model offers meaningful clinical utility for decision-making.

Sensitivity measures the model’s ability to accurately identify patients who actually experienced falls, reflecting the proportion of true positive cases correctly classified. Specificity represents the model’s capacity to distinguish patients without falls, indicating the proportion of true negative cases correctly identified. The Youden Index, an indicator evaluating the model’s overall ability to differentiate true patients from non-patients, is calculated as: Youden Index = (Sensitivity + Specificity) − 1. The optimal cutoff value is the threshold that maximizes the Youden Index, meaning it selects the point with the highest sum of sensitivity and specificity among all possible classification thresholds. In the training set, the Hosmer–Lemeshow test indicated good model fit (χ²=5.353, *P*=0.719), with a predictive accuracy of 83.7%. The area under the ROC Curve (AUC) was 0.832, and the maximum Youden index was 0.577, corresponding to a sensitivity of 74.7%, specificity of 83.0%, and an optimal cutoff value of 0.221. In the validation set, the model also demonstrated a good fit (χ²=5.678, *P*=0.128), with a predictive accuracy of 82.1%. The AUC was 0.793, with a maximum Youden index of 0.495, a sensitivity of 62.0%, a specificity of 87.5%, and an optimal cutoff value of 0.130. See **Figure 2**. The calibration curves further verified the model’s predictive accuracy and reliability. See **Figure 3**. Decision Curve Analysis (DCA) was drawn using R software to assess the clinical utility of the fall risk prediction model in elderly lung cancer patients with sarcopenia. In this study, the actual DCA decision curve was higher than the two extreme lines in most of the threshold probability range, indicating that our predictive model has some practical value in clinical decision making. See **Figure 4**.

## 4 Discussion

### 4.1 Analysis of fall risk factors in elderly lung cancer patients with sarcopenia

#### 4.1.1 Multiple metastases

Multiple metastases refer to the simultaneous formation of new tumors in multiple body sites by cancer cells. Compared with single-site metastasis, multiple metastases have more profound and widespread impacts on patients. Brain metastases may impair the nervous system, particularly the areas responsible for balance control, causing instability during walking or standing and increasing the risk of falls (25) below. Bone metastases often result in severe pain due to periosteal traction, soft tissue invasion, and nerve compression, which adversely affect sleep quality, daily living activities, and mobility, thus reducing walking ability (26, 27). Liver metastases may compromise metabolic and detoxification functions, leading to fatigue and reduced physical strength, making it difficult for patients to maintain balance during daily activities (28).

#### 4.1.2 Increased nocturia

This study revealed a strong correlation between nocturia and fall risk in elderly lung cancer patients with sarcopenia. Patients who experienced nocturia three or more times per night were six times more likely to fall than those with a normal frequency of nocturia. Several studies have also confirmed the significant association between nocturnal polyuria and falls (29–31).

#### 4.1.3 Sleep disorders

Symptoms of lung cancer, such as coughing, dyspnea, and chest pain, disrupt normal sleep patterns, causing frequent nighttime awakenings. Chronic sleep disturbances weaken patients’ physical strength and balance, increasing their susceptibility to falls during daily activities (32). Moreover, the psychological stress from a lung cancer diagnosis and treatment often induces anxiety and depression, further impairing sleep quality and contributing to daytime fatigue and helplessness, which heighten the risk of falls (33). Additionally, sleep disorders may negatively affect cognitive functions, including attention, memory, and judgment, making it difficult for patients to perceive environmental changes and respond appropriately while walking, thereby increasing the likelihood of falls (34, 35). Interventions to improve sleep quality, such as enhancing the sleep environment, providing psychological support, optimizing medication regimens, and strengthening health education, should be implemented to reduce fall risk.

#### 4.1.4 Frailty

This study identified frailty as a significant factor associated with falls in elderly individuals, which is consistent with findings from domestic and international studies (36–38). Sarcopenia, characterized by a progressive reduction in muscle fiber quantity and volume, leads to notable decreases in muscle strength, endurance, and coordination. These deficits severely impair walking and standing abilities, disrupt physical balance, and increase the risk of falls during daily activities (39). Healthcare providers must recognize the comprehensive threat frailty poses to patients’ overall health and quality of life. Comprehensive assessments of muscle strength, balance, and gait are crucial. Additionally, optimizing medication regimens, minimizing the use of fall-inducing drugs, and educating patients and their families about potential medication side effects are essential for preventing falls.

#### 4.1.5 Malnutrition

Malnutrition is a major risk factor for adverse clinical outcomes in cancer patients. It is one of the most common complications and persists throughout the disease course (40). Studies suggest that lung cancer patients often experience worse nutritional status than those with other cancers do (41). Li et al. reported a malnutrition incidence of 45.9% in lung cancer patients, with the incidence being three times greater in patients with sarcopenia (42). Furthermore, treatments for lung cancer, such as chemotherapy and radiotherapy, significantly impact the digestive system, causing symptoms such as anorexia, nausea, vomiting, and malabsorption, which exacerbate malnutrition (43). Combined with the metabolic abnormalities in glucose, fat, and protein often observed in lung cancer patients, these factors further weaken physical strength and balance (44).

### 4.2 Clinical application value of the fall risk prediction model for elderly patients with lung cancer and sarcopenia

This study focused on the prediction of falls, a common adverse clinical event, among lung cancer patients with sarcopenia. Using univariate and multivariate logistic regression analyses, the study systematically examined factors influencing fall risk. Five independent predictors were identified: multiple metastases, nocturia frequency ≥3 times, sleep disorders, frailty score, and malnutrition score. On the basis of these factors, a clinical prediction model was constructed and visualized via a nomogram. To ensure accuracy and reliability, the model was internally evaluated and validated. The results revealed that the areas under the ROC curve (AUCs) for the training and validation sets were 0.832 and 0.793, respectively, and the Hosmer–Lemeshow (HL) test results were 0.719 and 0.128, indicating excellent discriminative ability, calibration, and clinical utility. These findings suggest that the model offers robust predictive performance, providing scientific support for clinical predictions and improving fall risk management for patients. This study employed logistic regression to develop a risk prediction model, a method widely used in medical research due to its high computational efficiency and strong interpretability. However, its applicability may be limited when handling complex nonlinear features and high-dimensional interactions. Machine learning offers unique advantages in processing large-scale, high-dimensional medical data and has demonstrated significant potential in constructing disease risk prediction models (45). Future research could integrate AI/machine learning algorithms (e.g., random forests, neural networks) to further enhance predictive accuracy. Nevertheless, machine learning algorithms typically require larger sample sizes and computational resources, which exceed the scope of this single-center study with a small sample size. Additionally, this model did not incorporate biological indicators or image-based information.

## 5 Conclusion

The fall risk prediction model developed in this study, which is based on independent predictors, demonstrated strong predictive performance and is beneficial for healthcare providers in identifying high-risk patients. This study provides a scientific basis for formulating precise clinical management strategies. However, as a single-center study, the sample was drawn from a single hospital, limiting its representativeness and potentially affecting the generalizability of the findings. Future research should expand the sample size to increase model robustness and conduct external validations to ensure broader applicability and reliability.

## Data Availability

The original contributions presented in the study are included in the article/supplementary material. Further inquiries can be directed to the corresponding author.
